# Numerical Scheme for Compartmental Models: New Matlab Software Codes for Numerical Simulation

**DOI:** 10.12688/f1000research.130458.2

**Published:** 2023-09-25

**Authors:** Samuel Okyere, Joseph Ackora-Prah, Ebenezer Bonyah, Samuel Akwasi Adarkwa

**Affiliations:** 1 Mathematics, Kwame Nkrumah University of Science and Technology, Kumasi, Ghana; 2 Department of Mathematics and Applied Mathematics, University of Johannesburg, Johannesburg 2006, South Africa; 3 Mathematics Education, Akenten Appiah-Menka University of Skills Training and Enterpreneurial Development,, Kumasi, Ghana; 4 Department of Statistical Sciences, Kumasi Technical University, Kumasi, Ashanti Region, Ghana

**Keywords:** Compartmental Models, Numerical Simulation, Matlab software codes, R software codes

## Abstract

**Background:** This paper presents a newly developed Matlab code for the numerical simulation of compartmental/deterministic models. It addresses modeling and simulation issues concerning compartmental models. The code is easy to understand and edit for the simulation of compartmental models. An alternative codes for statistical software package R has been proposed for the same model. R software is freely available for use.

**Methods:** We proposed a basic SEIR model for illustration purposes. Matlab and R software codes are developed for the SEIR model which users can follow and easily understand the computations.

**Results:** The two codes work on all Matlab and R versions. For models with more compartments, we suggest using higher version of Matlab and R. Matlab works on windows, Mac and Linux

**Conclusions:** New Matlab software codes purposely for numerical simulations of classical deterministic models which can run on any version of Matlab has been introduced in this paper. This code can be edited/modify to suit any deterministic models and any desired output required. An alternative open source free version has been written in R has been provided as well

## Introduction

With the help of a programming language that represents matrix and array mathematics directly, MATLAB combines a desktop environment tailored for iterative analysis and design processes. ^1^ The Live Editor for writing scripts that mix code, output, and formatted text in an executable notebook is part of it. ^1^ The Windows requirements are Windows 10 (version 20H2 or higher), Windows 11, Windows Server 2019, and Windows Server 2022. Many scientists and mathematicians choose to use this program since it can be accessed across all popular platforms, including Linux, Mac, and Windows, and because it can be used to explore, model, and analyze data. ^1^ Matlab software has been used to run numerical simulations of compartmental models in epidemiology. ^2^^–^^11^

There are several fundamental compartmental models described using differential equations. The basic ones include Susceptible - Infected (SI), Susceptible - Infected - Recovered (SIR), ^12^^,^^13^ Susceptible - Infected - Susceptible (SIS), ^14^ Susceptible - Infected - Recovered - Vaccinated (SIRV), ^15^ Susceptible - Exposed - Infected - Recovered (SEIR) models. ^4^ The purpose of this study is to make public new Matlab codes that authors have been utilising in their work to aid researchers, especially students, who rely on deterministic or compartmental modeling of epidemiology in the numerical simulation of their research projects. This well-detailed code, in our opinion, might be extremely helpful to them as many of them struggle to do the numerical simulations due to the dearth of research that specifically tackles numerical simulation of deterministic models and also to provide users more freedom for coding in Matlab. Recently, researchers have started sharing their codes and providing detailed explanation on how to use them. To provide users extra coding freedom, Guo *et al.* ^16^ presented newly developed visualization framework called OpenSeesPyView, which is a Python programming-based graphical user interface (GUI) for OpenSeesPy, a prevalent finite element solver in earthquake engineering. The R package ag5Tools was written by Brown et al. ^17^ and offers a streamlined interface for downloading and retrieving AgERA5 data. With the help of the program, time-series data for groups of geographic points may be easily extracted and converted into a format that can be employed in statistical models used in agricultural research. The Rcall interface, developed by Egert and Kreutz, ^18^ gives users access to a large range of techniques written in MATLAB and R. The program is MATLAB-based and offers direct access to R-based tools and methodologies, such as those found on Bioconductor or CRAN. ShinyGAStool, an open source tool created by Hoffmann et al., ^19^ allows users to easily execute a candidate gene association analysis from a web browser using huge datasets. The remaining section are group as follows: The method section, where we demonstrate how to use the Matlab software codes. we look at the implementation, operation and discussions and limitations. The last section is the concluding section. The software section has the alternative software codes in R.

## Methods

### Implementation

**Figure 1.**
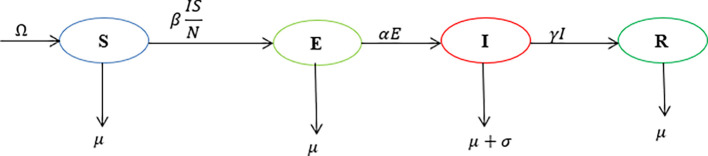
Model dynamics flowchart.

We demonstrate how to use the matlab software codes with the SEIR compartmental model depicted in Figure 1. The population is partitioned into four (4) compartments: Susceptible, exposed, infected and recovered. Individuals are recruited into the susceptible class at a rate Ω and they die at a rate μ . The transmission rate is β and the recovery rate is γ . The rate at which exposed individuals become infectious is α and the disease-induced death rate is σ .

The model is described by the following ordinary differential equations.

$$\begin{array}{l} \frac{\mathrm{dS}}{\mathrm{dt}}=\Omega -\beta \frac{\text{IS}}{N}-\mathrm{\mu S},\\ \frac{\mathrm{dE}}{\mathrm{dt}}=\beta \frac{\text{IS}}{N}-\left(\alpha +\mu \right)E,\\ \frac{\mathrm{dI}}{\mathrm{dt}}=\mathrm{\alpha E}-\left(\gamma +\sigma +\mu \right)I,\\ \frac{\mathrm{dR}}{\mathrm{dt}}=\mathrm{\gamma I}-\mathrm{\mu R}, \end{array}$$ (1)

with initial conditions $S\left(0\right)\geq 0,E\left(0\right)\geq 0,I\left(0\right)\geq 0,\;\text{and}\;R\left(0\right)\geq 0$ .

For the purposes of the simulations, the following parameter values are chosen and is given in Table 1. The initial conditions chosen are $S\left(0\right)=1000,E\left(0\right)=0,I\left(0\right)=1,R\left(0\right)=0$ .

**Table 1.** Parameter values and description.

Parameter	Description	Value	Source
Ω	Recruitment	29.08	^2^
β	Transmission rate	0.9	^2^
α	infectiousness of the exposed individuals	0.3	assumed
μ	natural rate of death	0.4252912 $\times 10^{-4}$	^2^
γ	Recovery rate of infected individuals	0.1	assumed
σ	Disease-induced death rate	0.003286	assumed

**Figure 2.**
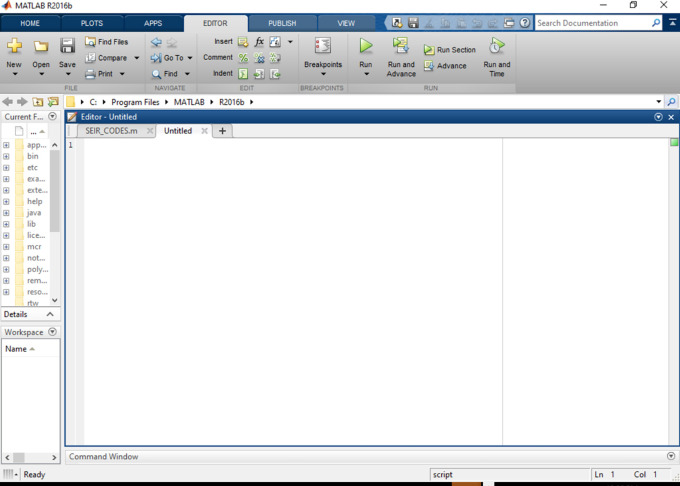
Matlab Editor window.

Once you have your model and parameter values clearly defined, you can then open the matlab editor window which is shown in Figure 2. Input or copy the codes and paste at the new script.

The numerical Matlab software codes used for model (1)

% It is the SEIR model Matlab software codes.

function [t,S,E,I,R]=SEIR_MODEL(Omega,beta,mu,sigma,alpha,gamma,N,S0,E0,I0,R0,MaxTime)

% where Omega=Ω, beta=β, mu=μ, sigma=σ, alpha=α,gamma=γ

### Operation



% Define the parameters
if nargin==0
  Omega=20;
  beta=0.3;
  mu=0.00004252912;
  alpha=0.3;
  sigma=0.003286;
  gamma=0.1;
%The initial condition
  S0=1000;
  E0=0;
  I0=1;
  R0=0.0;
  MaxTime=120;
end

**Figure 3.**
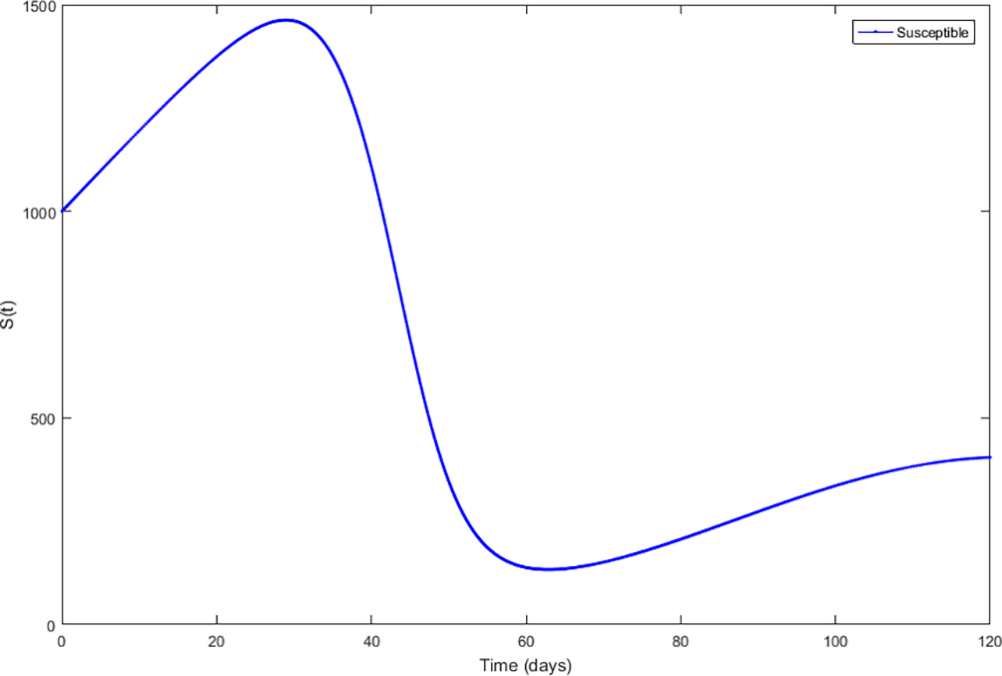
Susceptible compartment.

**Figure 4.**
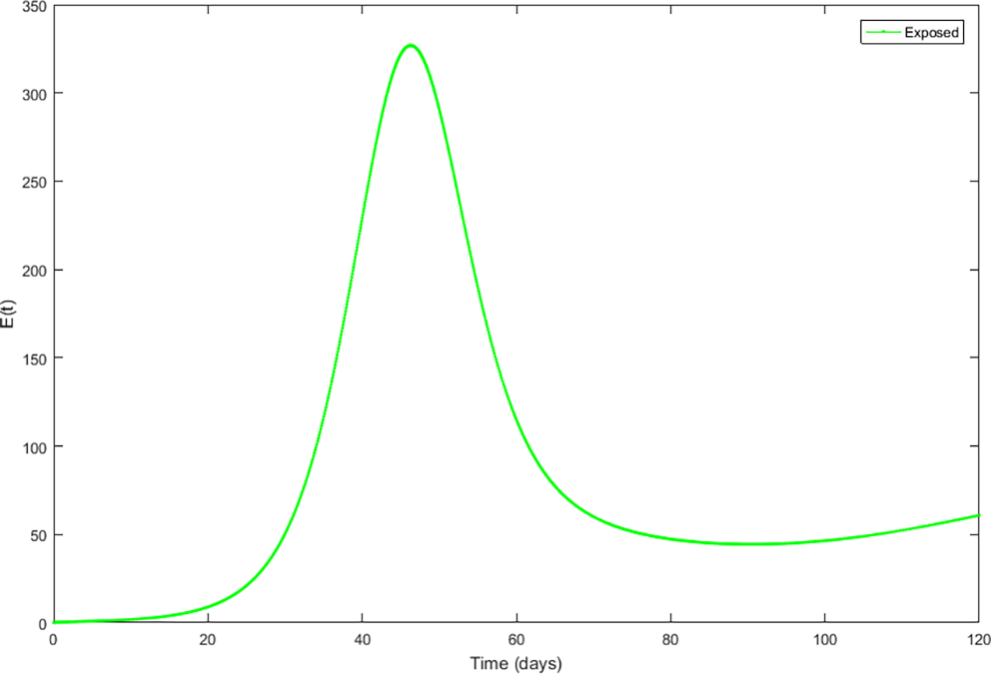
Exposed compartment.

**Figure 5.**
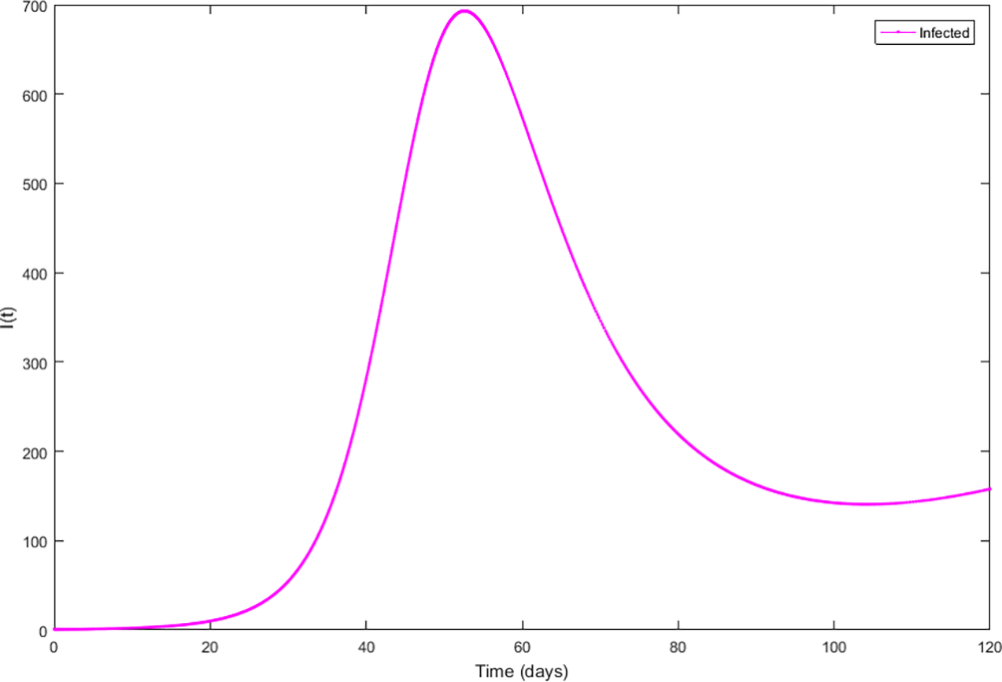
Infected compartment.

S=S0;E=E0;I=I0;R=R0;
N=S+E+I+R;
% The main iteration
options=odeset('RelTol',1e-5);
[t,pop]=ode45(@Diff_2_6,[0 MaxTime],[S E I R],options,[Omega gamma beta mu sigma alpha N]);

S=pop(:,1);E=pop(:,2);I=pop(:,3);R=pop(:,4);
% plots the graphs with scaled colours
% k=black colour, b=blue colour, g=green, m=mangetta
figure(1)
Y=plot(t,S,'b.');
legend(Y,'Susceptible')
xlabel 'Time (days)'
ylabel 'S(t)'

figure(2)
T=plot(t,E,'.g');
legend(T,'Exposed')
xlabel 'Time (days)'
ylabel 'E(t)'

figure(3)
f=plot(t,I,'.m');
legend(f,'Infected with Monkeypox')
xlabel 'Time (days)'
ylabel 'I(t)'

figure(4)
h=plot(t,R,'.g');
legend(h,'Recovered')
xlabel 'Time (days)'
ylabel 'R(t)'

figure(5)
W=plot(t,S,'.-b',t,E,'.-g',t,I,'.-m't,R,'.-k');
legend(W,'Recovered')
xlabel 'Time (days)'
ylabel 'R(t)'




**Figure 6.**
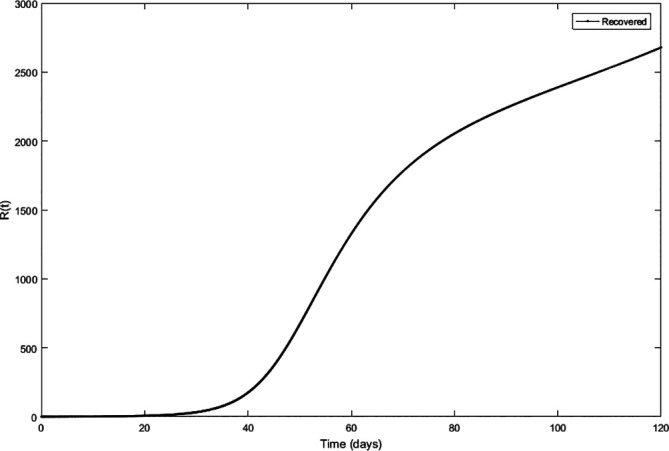
Recovered compartment.

**Figure 7.**
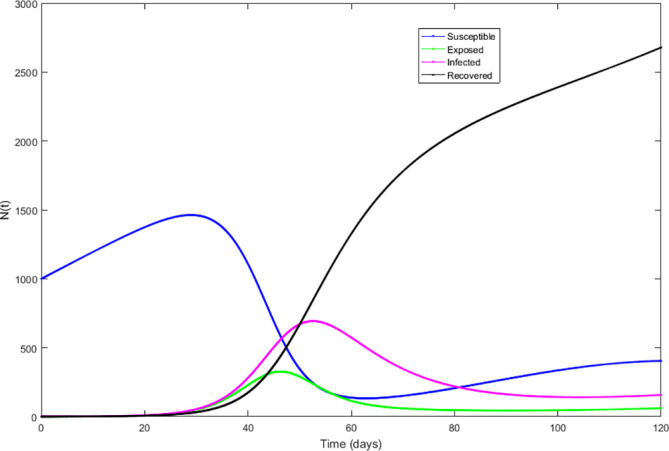
Dynamics of all compartments.

% calculates the differential rates used in the integration.
function dpop=Diff_2_6(t,pop, parameter)
Omega=parameter(1);gamma=parameter(2);beta=parameter(3);mu=parameter(4);sigma=parameter(5);alpha=parameter(6);N=parameter(7);
S=pop(1);E=pop(2);I=pop(3);R=pop(4);
dpop=zeros(4,1);
dpop(1)=Omega-(beta*I*S)./(N)-mu*S;
dpop(2)=(beta*(I)*S)./(N)-(alpha+mu)*E;
dpop(3)=alpha*E-(gamma+mu+sigma).*I;
dpop(4)=gamma*I-mu*R;


Upon running the codes, the simulation results are shown in Figures 3– 7.

## Discussion

**Figure 8.**
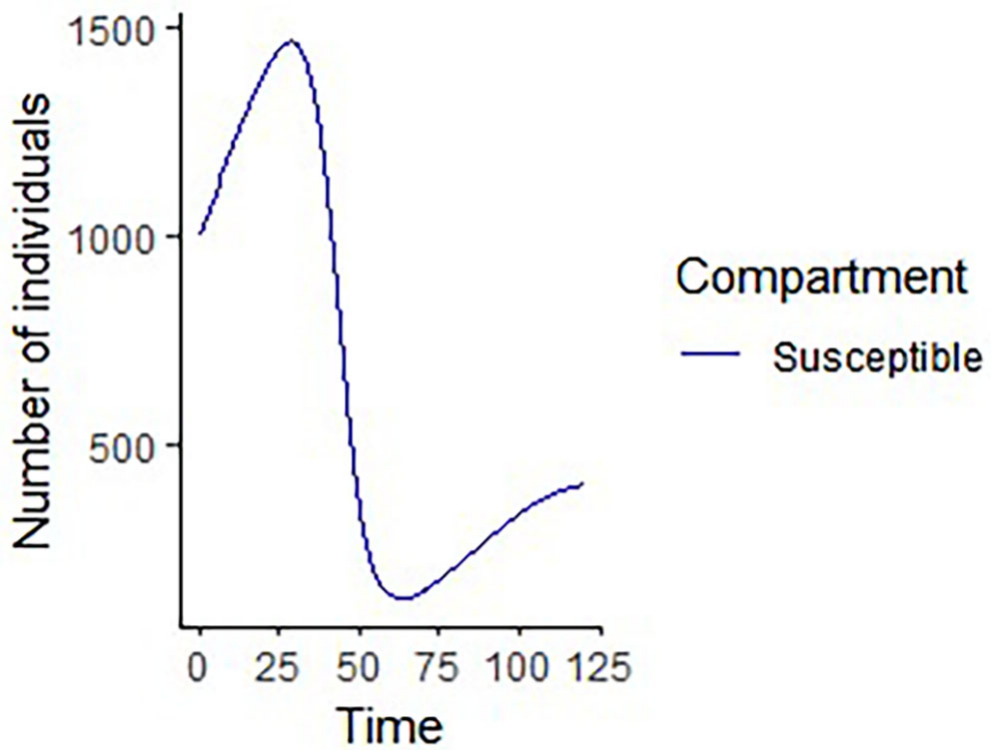
Susceptible compartment using R.

**Figure 9.**
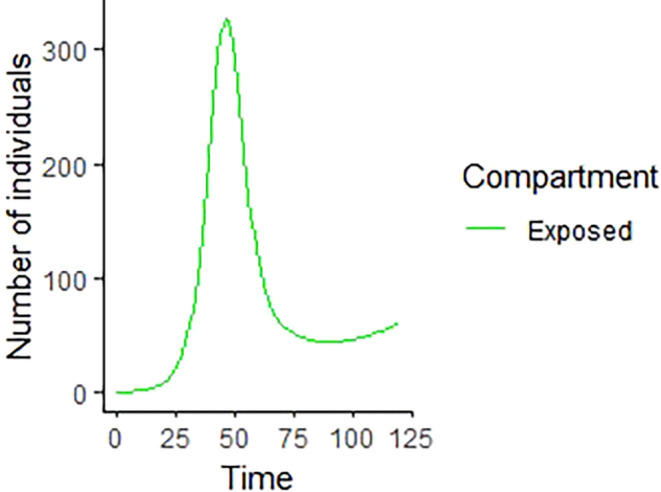
Exposed compartment using R.

**Figure 10.**
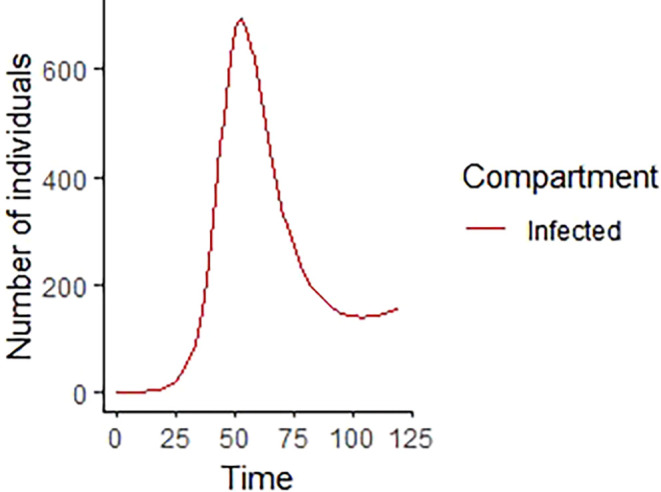
Infected compartment using R.

In Figures 3– 6, are reported, the numerical solutions of system (1) for a period of 120 days. These codes can be modified for any compartmental models. The ‘figure’ command produces the output given in Figures 3– 6. The steps or procedures listed in the codes have to be followed carefully in order not to encounter errors. The parameters can be represented with letters for instance Ω can be written in the codes as Omega as Matlab doesn’t recognize the parameters listed in the code. The code written in the editor window can be seen in Figure 7. The SEIR model is extended, and an alternative software, R codes has been provided at the appendix section. Using the same initial conditions and parameter values given in Table 1, the output figures for the R code are given by Figures 8– 10. Users who cannot afford Matlab software can freely use the R software codes for the numerical simulation. The two software codes gives the same output results.

% The R software codes

# Load required packages
library(deSolve)
library(ggplot2)
# Define the SEIR model function
SEIR_model <- function(time, state, parameters) {
 with(as.list(c(state, parameters)), {
 # Differential equations
 dS <- Omega - (beta * S * I) / N - S * mu
 dE <- (beta * S * I) / N - (alpha + mu) * E
 dI <- alpha * E - (gamma + sigma + mu) * I
 dR <- gamma * I - mu * R
 # Return the derivatives
 return(list(c(dS, dE, dI, dR)))
 })
}
# Define the model parameters
parameters <- list(
 Omega = 20, # Recruitment rate
 mu = 0.00004252912, # Death rate
 beta = 0.3, # Transmission rate
 gamma = 0.1, # Recovery rate
 alpha = 0.3, # Rate from exposed to infected
 sigma = 0.003286 # Disease-induced death rate
)

# Initial conditions
initial_state <- c(
 S = 1000, # Initial susceptible population
 E = 0, # Initial exposed population
 I = 1, # Initial infected population
 R = 0.0 # Initial recovered population
)
N = 1001

# Time points to solve the model
times <- seq(0, 120, by = 1)

# Solve the differential equations
out <- as.data.frame(ode(y = initial_state, times = times, func = SEIR_model, parms = parameters))

# Plot the results for Susceptible
ggplot(out, aes(x = time, y = value, color = variable)) +
 geom_line(aes(y = S, color = "Susceptible")) +
 labs(x = "Time", y = "Number of individuals") +
 labs(x = "Time", y = "Number of individuals", color = "Compartment") +
 scale_color_manual(values = c("blue")) +
 theme_classic()

# Plot the result for Exposed
ggplot(out, aes(x = time, y = value, color = variable)) +
 geom_line(aes(y = E, color = "Exposed")) +
 labs(x = "Time", y = "Number of individuals") +
 labs(x = "Time", y = "Number of individuals", color = "Compartment") +
 scale_color_manual(values = c("green")) +
 theme_classic()

# Plot the result for Infected
ggplot(out, aes(x = time, y = value, color = variable)) +
 geom_line(aes(y = I, color = "Infected")) +
 labs(x = "Time", y = "Number of individuals") +
 labs(x = "Time", y = "Number of individuals", color = "Compartment") +
 scale_color_manual(values = c("red")) +
 theme_classic()

# Plot the results for recovered
ggplot(out, aes(x = time, y = value, color = variable)) +
 geom_line(aes(y = R, color = "Recovered")) +
 labs(x = "Time", y = "Number of individuals") +
 labs(x = "Time", y = "Number of individuals", color = "Compartment") +
 scale_color_manual(values = c("black")) +
 theme_classic()

# Plot the results for all compartments
ggplot (out, aes(x = time)) +
 geom_line(aes(y = S, color = "Susceptible")) +
 geom_line(aes(y = E, color = "Exposed")) +
 geom_line(aes(y = I, color = "Infected")) +
 geom_line(aes(y = R, color = "Recovered")) +
 labs(title = "SEIR Model",
  x = "Time",
  y = "Population",
  color = "Compartment") +
 scale_color_manual(values = c("Susceptible" = "blue", "Exposed" = "green", "Infected" = "red", "Recovered" = "black"))+
 theme_classic()


**Figure 11.**
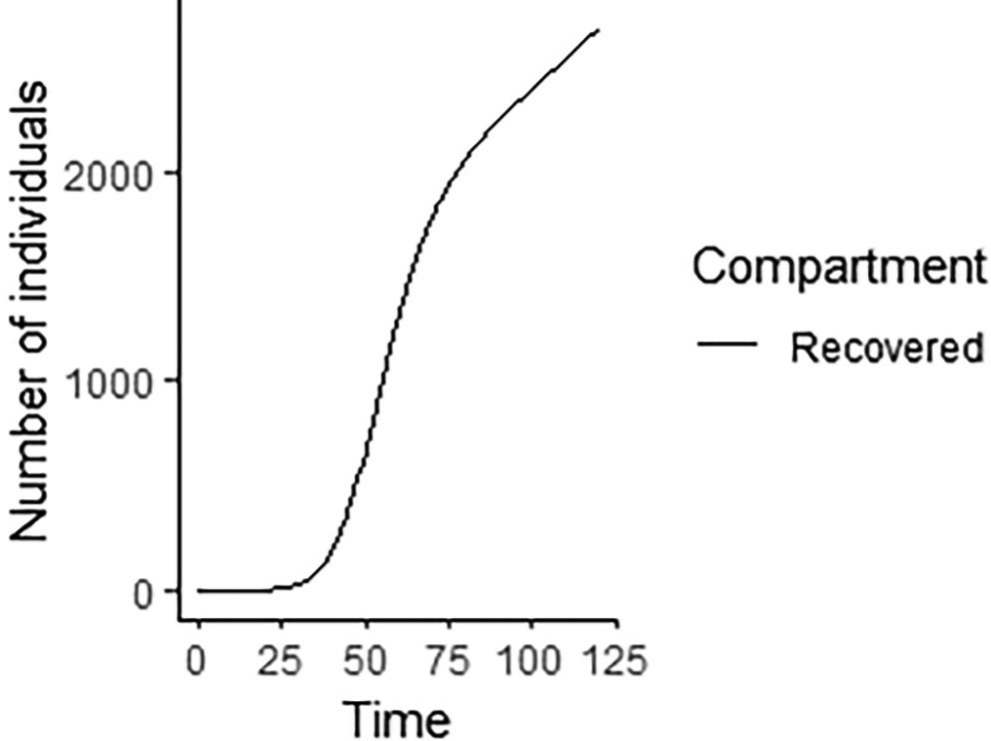
Recovered compartment using R.

**Figure 12.**
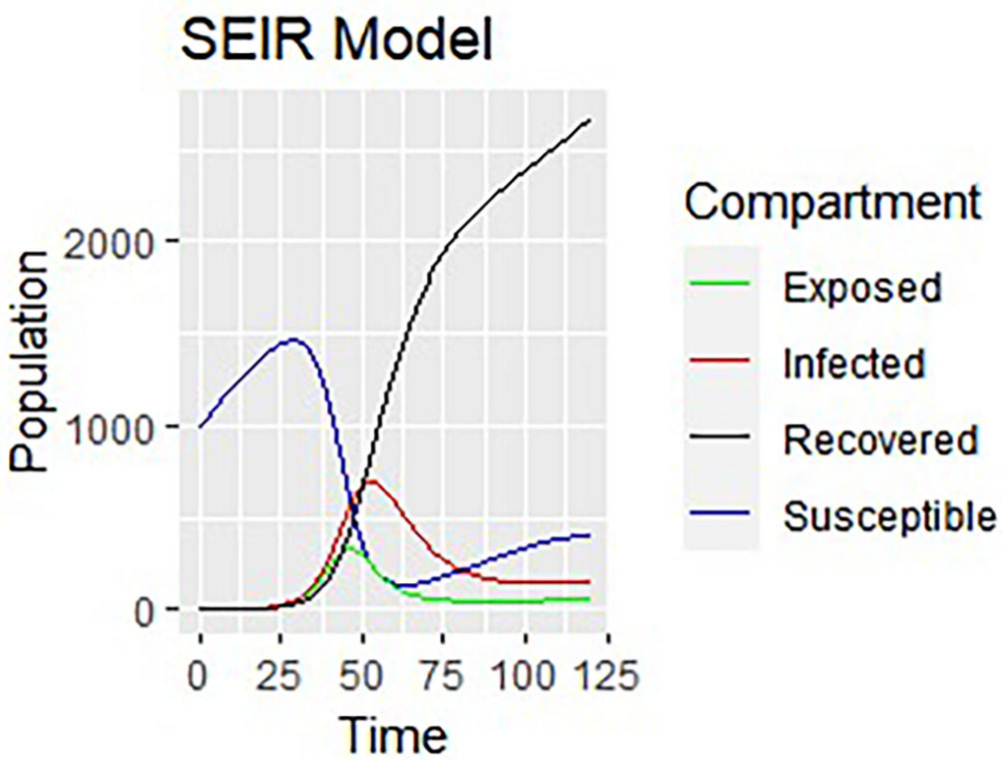
All the compartments using R.

% The results of the numerical simulation gives Figures 8- 12.

### Limitations

MATLAB can disable some advanced graphics rendering features by switching to software OpenGL by ignoring extra legend entries.

R on the other hand, has a limited memory capacity which can be a problem when working with large data sets or running computationally-intensive analyses. It can be relatively slow compared to other programming languages like Matlab, C++ or Python, especially for certain types of calculations. It also has limited graphical capabilities.

## Conclusion

This work seeks to introduce new matlab software codes purposely for numerical simulations of classical compartmental models which can run on any version of Matlab. The intended targets are researchers and students who uses Matlab for their analysis. These codes can be edited/modify to suit any deterministic models and any desire output required. The SEIR deterministic model was used to give a much insight about the codes. Alternatively, a deterministic SEIR codes written in R software is provided for those who wants to use freely available software. Despite the limitations of the R software, the deterministic model implemented in the R code can still be a useful tool for understanding the basic dynamics of disease transmission.

## Data Availability

OSF: Raw_data_monkeypox. DOI: 10.17605/OSF.IO/2J5R9. This project contains the following underlying data: Data_used.pdf (data input into matlab simulations) OSF: Output_data DOI: 10.17605/OSF.IO/7EJUP. This project contains the following extended data: MATLAB Command Window1.pdf (Matlab output data that accompanied the numerical simulation figures) Data are available under the terms of the Creative Commons Zero “No rights reserved” data waiver (CC0 1.0 Public domain dedication). Source code available from: https://github.com/okyere2015/Matlab_codes/releases/tag/v2.0.1. Archived source code at the time of publication: https://doi.org/10.5281/zenodo.7671815. License: Apache 2.0
