# Supplementary material for: Numerical Scheme for Compartmental Models: New Matlab Software Codes for Numerical Simulation
Source: F1000Res. 2023 Sep 25;12:445. Originally published 2023 Apr 26. [Version 2] doi: 10.12688/f1000research.130458.2 (PMC10579850; doi:10.12688/f1000research.130458.2)
Supplement: Peer review history

# Reviewer report: Reviewer response for version 2

**DOI:** 10.5256/f1000research.155959.r209183

**Published:** 2023-10-17

**Authors:** Shewafera Wondimagegnhu Teklu

**Affiliations:** 1 Department of Mathematics, Debre Berhan University, Debre Birhan, Amhara, Ethiopia

I have read the author's revised manuscript and since the authors addressed the given comments the revised manuscript can be indexed.

Are the conclusions about the tool and its performance adequately supported by the findings presented in the article?

Partly

Is the rationale for developing the new software tool clearly explained?

Partly

Is the description of the software tool technically sound?

Yes

Are sufficient details of the code, methods and analysis (if applicable) provided to allow replication of the software development and its use by others?

Partly

Is sufficient information provided to allow interpretation of the expected output datasets and any results generated using the tool?

Partly

Reviewer Expertise:

Numerical Analysis, Mathematical Modelling, Mathematical Biology and Epidemiology.

I confirm that I have read this submission and believe that I have an appropriate level of expertise to confirm that it is of an acceptable scientific standard.

# Reviewer report: Reviewer response for version 2

**DOI:** 10.5256/f1000research.155959.r209184

**Published:** 2023-10-17

**Authors:** Belela Samuel Kotola

**Affiliations:** 1 Department of Mathematics, Oda Bultum University, Asebe Teferi, Oromia, Ethiopia

The article has been revised in requested manner. 

 Therefore, I would like to recommend  an acceptance for this article.

Are the conclusions about the tool and its performance adequately supported by the findings presented in the article?

Yes

Is the rationale for developing the new software tool clearly explained?

Yes

Is the description of the software tool technically sound?

Yes

Are sufficient details of the code, methods and analysis (if applicable) provided to allow replication of the software development and its use by others?

Yes

Is sufficient information provided to allow interpretation of the expected output datasets and any results generated using the tool?

Partly

Reviewer Expertise:

Mathematical modeling

I confirm that I have read this submission and believe that I have an appropriate level of expertise to confirm that it is of an acceptable scientific standard.

# Reviewer report: Reviewer response for version 1

**DOI:** 10.5256/f1000research.143219.r188924

**Published:** 2023-09-04

**Authors:** Shewafera Wondimagegnhu Teklu

**Affiliations:** 1 Department of Mathematics, Debre Berhan University, Debre Birhan, Amhara, Ethiopia

Comments to the Authors

 Title: Numerical Scheme for Compartmental Models: New Matlab Software Codes for Numerical Simulation

 The authors proposed new Matlab Software Codes for Numerical Simulation and also an alternative codes for statistical software package R has been proposed for the same compartmental model.

  

1. In their discussion section the authors written as the SEIR model is extended, and alternative software, R codes has been provided at the appendix section. In their manuscript I did not find the appendix section and I have seen only the MATLAB code to illustrate the proposed SIER model. Give the R code on the revised manuscript!
2. The authors needs to disclose the numerical codes using MATLAB and R software for potential new researchers and possibly students but they should refine the two codes separately and compare and contrast them with the proposed trivial SIER model.
3. I do not think, with these current details, that the manuscript can be accepted. It needs a modification.

Are the conclusions about the tool and its performance adequately supported by the findings presented in the article?

Partly

Is the rationale for developing the new software tool clearly explained?

Partly

Is the description of the software tool technically sound?

Yes

Are sufficient details of the code, methods and analysis (if applicable) provided to allow replication of the software development and its use by others?

Partly

Is sufficient information provided to allow interpretation of the expected output datasets and any results generated using the tool?

Partly

Reviewer Expertise:

Numerical Analysis, Mathematical Modelling, Mathematical Biology and Epidemiology.

I confirm that I have read this submission and believe that I have an appropriate level of expertise to confirm that it is of an acceptable scientific standard, however I have significant reservations, as outlined above.

# Reviewer report: Reviewer response for version 1

**DOI:** 10.5256/f1000research.143219.r200352

**Published:** 2023-09-04

**Authors:** Belela Samuel Kotola

**Affiliations:** 1 Department of Mathematics, Oda Bultum University, Asebe Teferi, Oromia, Ethiopia

First and for most I would like to acknowledge the editor for giving me this chance for reviewing the article. Depend up on my back ground knowledge I have reviewed the article as follow.

 The researcher has been defined MATLAB as a programming language that represents matrix and array mathematics directly, providing a desktop environment for iterative analysis and design processes. Moreover, the area where it is widely used was described by the authors so that to be by scientists and mathematicians for exploring, modeling, and analyzing data across various platforms.

 The study has been aimed to make public new Matlab codes that authors have been using to aid researchers in numerical simulations of deterministic or compartmental models in epidemiology; which is the fundamental and timely objectives. Researchers have also started sharing their codes and providing detailed explanations on how to use them.

 This work introduces an editable and modified Matlab software codes for numerical simulations of classical compartmental models, suitable for researchers, students and for those seeking freely available software.

 Finally I recommend the article to be indexed after the following question and recommendation are addressed.

 RECOMMENDATION:

1. Some of the graphs can be plotted in a single window, so I recommend the authors plot some of the graphs, such as susceptible class, infected class, and exposed class, in a single window editor. Plotting in such a way can help us compare the relationships and differences among the plotted classes.
2. An author has stated the disadvantage of MATLAB as it disables advanced graphics rendering features by using OpenGL, and while R also has limited memory capacity, slowness, and limited graphical capabilities, making it suitable for large data sets and computationally intensive analyses, which one is the best? As an author, which software do you recommend to researchers? For what reason do you recommend it?

Are the conclusions about the tool and its performance adequately supported by the findings presented in the article?

Yes

Is the rationale for developing the new software tool clearly explained?

Yes

Is the description of the software tool technically sound?

Yes

Are sufficient details of the code, methods and analysis (if applicable) provided to allow replication of the software development and its use by others?

Yes

Is sufficient information provided to allow interpretation of the expected output datasets and any results generated using the tool?

Partly

Reviewer Expertise:

Mathematical modeling

I confirm that I have read this submission and believe that I have an appropriate level of expertise to confirm that it is of an acceptable scientific standard, however I have significant reservations, as outlined above.
